# The Dual Effect of Cannabinoid Receptor-1 Deficiency on the Murine Postoperative Ileus

**DOI:** 10.1371/journal.pone.0067427

**Published:** 2013-07-03

**Authors:** Yong-yu Li, Ming-hua Cao, Brigitte Goetz, Chun-qiu Chen, Ya-jing Feng, Chang-Jie Chen, Michael S. Kasparek, Andrej Sibaev, Martin Storr, Martin E. Kreis

**Affiliations:** 1 Department of Pathophysiology, Institute of Digestive Disease, Tongji University School of Medicine, Shanghai, China; 2 Department of Surgery, Hospital Grosshadern, Ludwig-Maximilian's University, Munich, Germany; 3 Department of Internal Medicine II, Hospital Grosshadern, Ludwig-Maximilian's University, Munich, Germany; 4 Charité University Medicine, Department of General-, Visceral- and Vascular Surgery, Campus Benjamin Franklin, Berlin, Germany; National Institute for Viral Disease Control and Prevention, CDC, China

## Abstract

**Introduction:**

Intestinal inflammatory responses play a critical role in the pathogenesis of postoperative ileus (POI). As cannabinoid receptor-1 (CB1) is involved in inhibiting gastrointestinal (GI) motility and anti-inflammation, we aimed to explore its contribution to POI.

**Methods:**

Experimental POI was induced in adult female CB1-deficient (CB1–/–) mice and wild-type littermates (C57BL/6N) by standardized small bowel manipulation. Twenty-four hours after surgery, GI transit was assessed by charcoal transport. FITC avidin, F4/80, and myeloperoxidase immunohistochemistry techniques were used to evaluate the inflammatory response in the muscularis of ileum and colon. Expressions of p38MAPK and its phosphorylated form (pp38) in the intestine were determined. Plasma levels of proinflammatory cytokines and chemokines were measured by ELISA as well.

**Results:**

POI was characterized by decreased GI transit (p<0.01) and accompanied by a marked intestinal and systematic inflammatory response in wild-type and CB1–/– mice. Increased numbers of inflammatory cells, including macrophages, neutrophils, and mast cells were observed in the muscularis of ileum and colon (p<0.01, or p<0.05). Plasma levels of interleukin-6 (IL-6), cytokine-induced neutrophil chemoattractant-1 (CINC-1/KC), and monocyte chemoattractant protein-1 (MCP-1) were elevated (p<0.01, or p<0.05). Expression of p38 and pp38 increased in the intestine (p<0.01, or p<0.05). CB1–/– mice showed an increased inflammatory response during POI, especially the systemic inflammatory markers, such as IL-6, KC, CINC1, and pp38 expression were increased as compared to those in WT mice (p<0.05).

**Conclusions:**

Intestinal motility was inhibited during POI. In this condition, inhibition of motility did not seem to be altered by the absence of CB1 receptors, however, an increased inflammatory response was observed in CB1–/– mice. Hence, CB1 receptor activation rather than inhibition may reduce the inflammatory response in POI, which has a remote potential to relate into reduced inhibition of intestinal motility during POI.

## Introduction

Postoperative ileus (POI) is defined as inhibition of gastrointestinal(GI) motility after abdominal surgery, which is one of the commonly seen postoperative disorders in surgical departments. The majority of these patients show mild symptoms, and recover in 2–3 days without medical intervention. Nevertheless, some patients have GI motility disorders lasting for a long time, which are difficult to treat, and therefore incur prolonged hospitalization and escalated medical costs [1], [2].

Many studies have been conducted on POI pathogenesis, yet the complete mechanism has not been fully elucidated. It is generally accepted that development of POI is multi-factorial and stems from failure of neurohumoral regulation of GI motility. This may be caused by anesthesia and mechanical bowel manipulation during surgery [3], [4]. The subsequent imbalance of the sympathetic – parasympathetic nervous system, i. e. sympathetic overactivity, cause POI [5], [6]. Since the late 1990's, the theory that inflammation plays an important role in the pathogenesis of POI has been supported by increasing experimental evidences. In POI animal models, scientists have observed inflammatory responses characterized by leukocyte infiltration in the intestinal muscularis, and elevated levels of inflammatory mediators in tissues and plasma 24 h after abdominal surgery [2], [7], [8]. Kalff et al. [8] demonstrated the increased mRNA and protein expression of intercellular adhesion molecule 1 (ICAM-1) and p-selectin in the intestinal muscularis of POI, and the introduction of ICAM-1 antibody may prevent the aggregation of monocytes and neutrophils in the intestinal muscularis and ameliorate the functional disorder of jejunum circular muscle during POI. In the previous work, we confirmed this inflammatory response in the intestinal muscularis, and showed elevated myeloperoxidase (MPO) activity indicating increased numbers of neutrophils during POI [9]. All of these studies explored the role of inflammatory responses in POI at its early stage, few hours after the surgical operations [10].

The cannabinoid system is involved in GI motility and secretion [11], [12]. In keeping with these observations, cannabinoid receptor-1 (CB1) was shown to be localized in the GI tract of many species, including humans [11]–[15]. CB1 was also shown to be present in neurons of the myenteric and submucosal plexus of the ileum and the colon [16]. Activation of CB1 reduces electrically induced contractions and movements [17], [18] and slows motility throughout the gut [19], [20]. In addition, the anti-inflammatory potential of cannabinoids has been of interest since their discovery in mammalians [16]. Enhancement of cannabinoid signaling and increased expression of CB1/CB2 receptors and/or endocannabinoid levels were observed following inflammatory stimuli in animals and in intestinal biopsies from patients with gut inflammatory disorders [21]–[23]. Several groups also showed that cannabinoids had exerted anti-inflammatory actions in the gut by activating CB1 receptor, and that the mechanism of action had involved inhibition of chemokines and proinflammatory cytokines, which were mainly released from macrophage and mast cells [24], [25].

Considering that CB1 activation slows GI motility and possesses anti-inflammatory potential as well, we aimed to investigate the involvement and role of CB1 in POI and the possible mechanisms. Specifically, we hypothesized that intestinal and systemic inflammatory responses associated with POI were increased in CB1-deficient mice [26], and design a study to elucidate whether activation of CB-1 receptors may serve as a potential target for prevention or treatment of POI.

## Methods

### Model of Postoperative Ileus

Adult female CB1-deficient (*CB1–/–*) mice and wild-type littermates (body weight of 25–35 g) in C57BL/6N background as described previously [26] were used in this study. These mice were kept in-house for at least 1 week prior to experiments. Before and during the experiments the animals were housed and maintained under controlled environmental conditions: in plastic sawdust floor cages at constant temperature (22°C) and a 12∶12-h light–dark cycle with free access to standard laboratory chow and tap water. The animal experiments were carried out in accordance with the national and international guidelines as outlined in the Guide for the Care and Use of Laboratory Animals, using the protocols approved by the Government of Bavaria animal use committee.

According to previously published procedures [9], postoperative ileus was induced. In brief, under enflurane anesthesia, animals were laparotomized and followed by sham treatment or standardized small bowel manipulation. The small bowel was pulled out gently onto moist gauze, and systematically manipulated from the ligament of Treitz to the terminal ileum for 5 min with two moist cotton applicators to induce POI. Control mice received sham operation without bowel manipulation. The laparotomy was closed with a running suture and all animals recovered quickly from surgery and generally began to eat and drink within several hours after surgery.

### Determination of Intestinal Transit and Sampling

GI transit and inflammatory responses of POI were investigated at 24 h time after surgery. GI transit was measured as described previously [23]. Briefly, mice were given a black marker (10% charcoal suspension in 10% gum arabic, 0.1 mL per 10 g body weight) administered orally. After 20 min, mice were sacrificed by enflurane inhalation and subsequent cervical dislocation. Blood samples were collected by cardiac puncture, and the small intestine was removed immediately from the pylorus to the cecum. The distance travelled by charcoal in the intestine was determined in centimeters and expressed as a percentage of total length of small intestine. Immediately afterwards, segments of terminal ileum and colon were harvested individually for histological and immunohistochemistry workup. Blood samples were kept in heparinized tubes and centrifuged for 10 min at 12,000 g, 4°C. Plasma samples were stored at −80°C until future use.

### Histological Evaluation of Gut

In order to investigate morphological changes of the gut during POI, histological evaluation was performed on ileum and colon samples. After washing with normal saline (NS), intestinal samples were fixed in 4% paraformaldehyde over night and embedded in paraffin. Thereafter, tissue slices (5 μm) were stained with hematoxylin and eosin (HE), and evaluated under a microscope.

### Inflammatory Cell Evaluation in Intestinal Smooth Muscle

Inflammatory cells i.e. mast cells, macrophages, monocytes and neutrophils were visualized by FITC avidin staining, F4/80 staining, and myeloperoxidase staining. Histological workup was performed on whole mounts of mouse intestinal muscularis to determine the extent of postoperative intestinal inflammation. Separate segments of ileum and colon were washed with cold Krebs Ringer solution (pH 7.4). Mucosa and submucosa were removed, and the muscularis layer was stretched 150% in length and 250% in width, followed by fixing in 100% ethanol for 10 minutes. Staining was performed with whole mounts, and procedures included in detail:

1. FITC avidin staining: whole mounts were incubated over night in FITC Avidin (1∶10) in 10% normal horse serum (NHS) at 4°C and mounted in quick-hardening Eukitt medium.
2. F4/80 staining: staining was performed by pre-incubation of whole mounts in 10% normal goat serum (NGS) in PBT for 1h at room temperature followed by an incubation with primary antibody (F4/80, rat anti-mouse MCA 497 1∶1 in PBS) overnight at 4°C. Specimens were incubated with secondary antibody (donkey anti-rat alexa fluor 488, 1∶100 in PBS) for 1 h at room temperature and were mounted thereafter in quick-hardening Eukitt medium.
3. Myeloperoxidase (MPO) staining: as described previously [9], staining of MPO-positive cells was performed by incubating whole mounts in a mixture of 10 mg Hankers-Yates reagent, 10 ml Krebs-Ringer buffer, and 100 μL 3% hydrogen peroxidase for 10 minutes. To quantify FITC avidin positive cells or F4/80 staining, a fluorescent microscope (Axiophot, Zeiss, Feldbach, Switzerland) was used at a 400 fold magnification. MPO staining was evaluated with a light fluorescence microscope (BX41, Olympus, Essex, UK) at a 200 fold magnification. Cells were counted in 15 randomly chosen areas with 5 fields in horizontal direction, 5 fields in vertically direction and 5 fields in diagonal direction for each specimen. Evaluation was repeated in 6 mice in each group. Counts are given as positive cells per square millimeter (the cell count/mm^2^).

### Determination of Cytokine and Chemokine in Mouse Plasma by ELISA

Plasma levels of cytokines and chemokines were determined by commercially available mouse-specific enzyme-linked immunosorbent assay (ELISA) kits for TNF-α, IL-6, cytokine-induced neutrophil chemoattractant-1 (CINC-1/KC) and monocyte chemoattractant protein-1 (MCP-1) based on the protocols provided by the manufacturer. Each sample was measured in duplicate using a microplate reader, and data are expressed as pg/ml plasma or percentage of controls.

### Examination of p38MAPK by Immunohistochemistry

The expression and localization of p38MAPK, a family member of the mitogen-activated protein kinases (MAPKs), and its phosphorylated form (pp38) in ileum and colon of mice were determined by immunohistochemistry. After conventional pretreatment, the slides with sections of mouse intestinal tissues were incubated overnight at 4°C in a humidified chamber with the related anti-p38 or anti-pp38 polyclonal antibody (1∶25 or l∶50 dilution, respectively). 50 µl of biotin-labeled goat anti-rabbit IgG working fluid was applied onto each slide and incubated at 37°C for 15 min, followed by incubation with a HRP labeled streptavidin working solution at 37°C for 15 min. Finally, the slides were DAB-stained and nuclear re-stained with hematoxylin. The negative control group was carried out through the same steps as described above, but the primary antibody was replaced by PBS. Positive staining was represented by brown dyeing in the sections, and image analysis of semi-quantitative data from five sections for each specimen was accomplished using digital Motic Med 6.0 system (Motic, Germany).

### Reagents and ELISA Kits

Enflurane was purchased from Abbott (Wiesbaden, Germany), mounting medium from Kindler (Freiburg, Germany), charcoal/gum arabicum, FITC avidin, hydrogen peroxide, and normal goat serum/normal horse serum from Sigma-Aldrich (Steinheim, Germany), F4/80 antibody (MCA 497) from AbD Serotec (Oxford, UK), donkey anti-rat Alexa 488 from Invitrogen (Eugene, Oregon, USA), and Hanker Yates reagent from Polysciences (Warrington, PA, USA). ELISA kits (MTA00, M6000B, MKC00B, MJE00) for determination of TNF-α, IL-6, CINC-1, MCP-1 were purchased from R&D Systems (Minneapolis, USA). Anti-p38 or anti-pp38 polyclonal antibody (SC-728 and SC-101758) were obtained from Santa Cruz (California, USA), biotin-labeled goat anti-rabbit IgG and HRP labeled streptavidin working solution from Biosynthesis Biotechnology (Beijing, China), and DAB staining kit from Boster Biological Technology (Wuhan, China).

### Data Analysis

Data are expressed as mean values ± SEM or mean values ± SD as indicated in the figure legends. All data were analyzed by using one-way ANOVA followed by Tukey's multiple comparison with SPSS 13.0 software (SPSS Co. Ltd., Shanghai, China). Values of *P*<0.05 were considered statistically significant. N in every group indicates the number of independent observations. Evaluations of all parameters were performed in a blinded fashion wherever technically possible.

## Results

### Intestinal Motility

**Figure 1:**
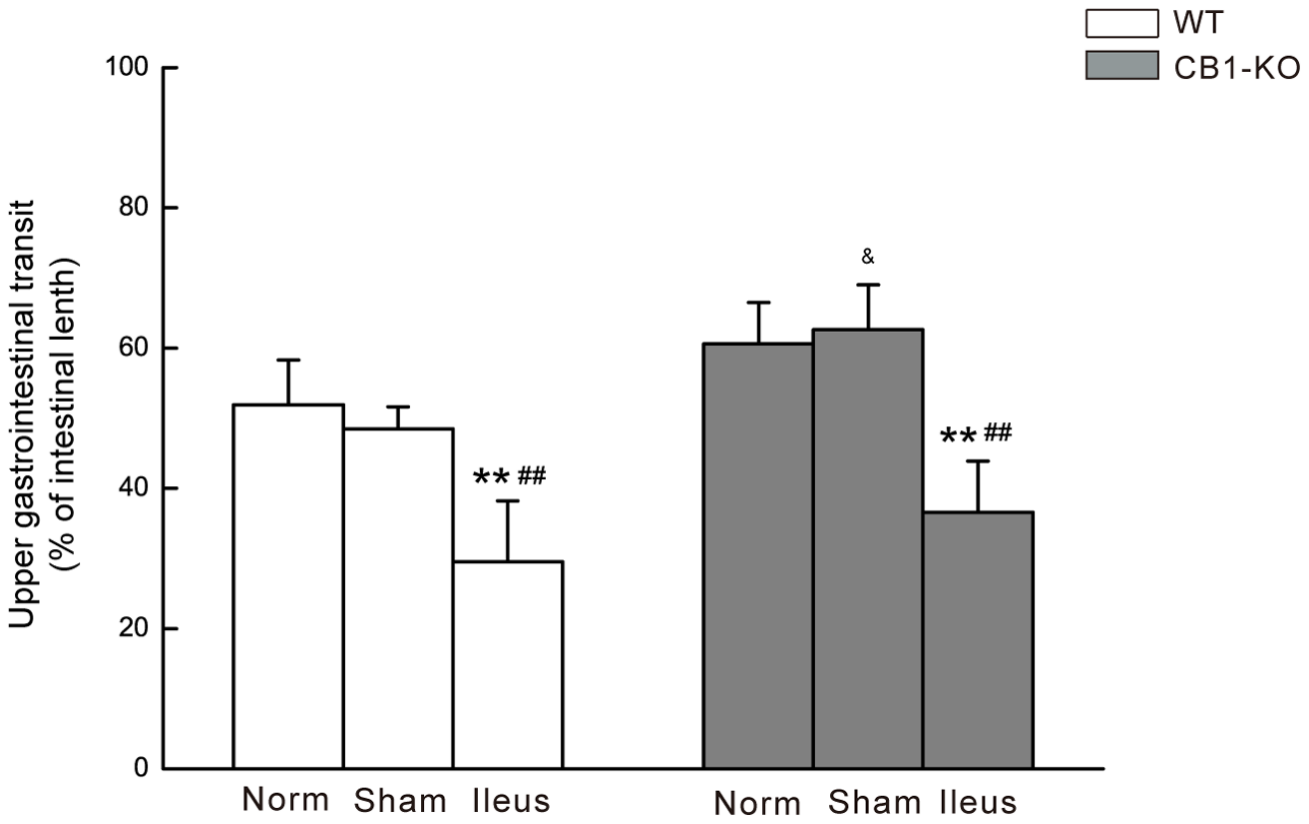
Upper GI transit in WT and *CB1–/–* (CB1-KO) mice. Gastrointestinal transit is determined as the distance travelled by orally-administered charcoal and presented as the percentage of total length of small intestine. Data are mean ± SD (n = 6/group). **P<0.01 vs. Control; ^##^ *P*<0.01 vs. Sham group; and & *P*<0.05, CB1–/– vs. identically-treated groups in WT mice.

Upper GI transit was not affected by sham operation (*P*>0.05) but significantly reduced at 24 h of POI in *CB1–/–* and WT mice (both *P*<0.01 compared to controls and sham operated groups). *CB1–/–* mice had a relatively faster GI transit in percentage of the intestinal length than that of WT mice in all treatment groups as detailed in Fig. 1.

### Histological Changes of Ileum and Colon

**Figure 2:**
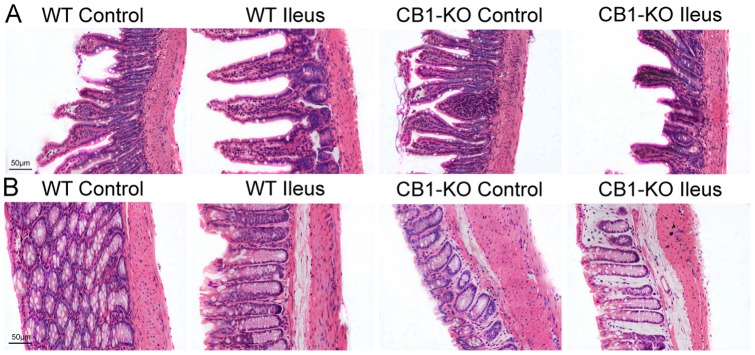
Histological changes in intestinal tissues of mice. A shows ileum tissue, and B shows colonic tissue sections from WT and *CB1–/–* (CB1-KO) mice. Excised ileum and colon segments were paraffin embedded, sliced, and stained with hematoxylin and eosin (HE), and observed under a microscope (original magnification 100×). Scale bar  = 50 µm.

As shown in Fig. 2, no obvious structural lesions were found in intestinal tissues at 24 h of POI under light microscopy. When compared with sham operated controls, edema and immune cells were observed in the submucosa of ileum and colon during POI in both types of mice (Fig. 2A and 2B).

### Immunohistochemistry of Inflammatory Cells in the Muscularis of Ileum and Colon

**Figure 3:**
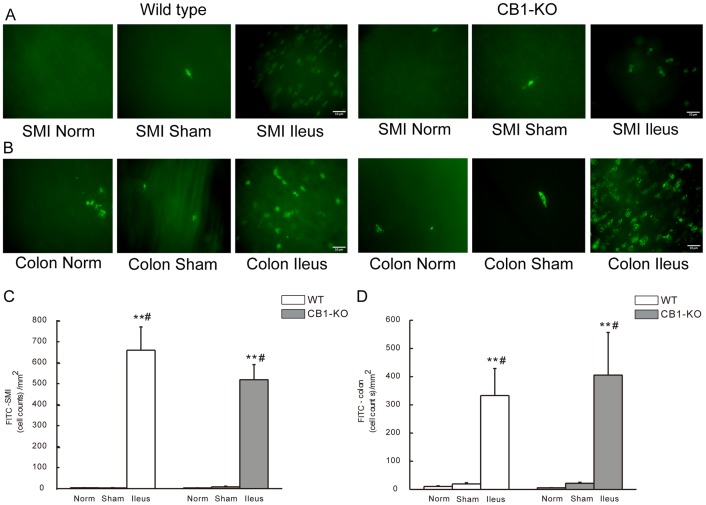
FITC avidin staining for mast cells in whole mounts of intestinal muscularis of mice. A and B show representative staining figures of FITC-avidin positive cells in small intestine (SMI) (A) and in colon (B) from WT or *CB1–/–* mice. C and D show statistical histograms of FITC-avidin positive cells in SMI (C) and in colon (D). The given cell counts are as positive cells per square millimeter (mean ± SEM, n = 6). **P<0.01 vs. normal controls, #*P*<0.05 vs. sham operated mice. Scale bar  = 10 µm.

At 24 h of POI, numbers of FITC-avidin positive cells (i. e. mast cells) were increased per square millimeter in the muscularis layer of ileum (Fig. 3A) or colon (Fig. 3B) in both WT and in CB1–/– mice (Fig. 3A–3D). In normal or sham-control mice, fewer FITC-avidin positive cells were found in the muscularis layer compared to POI mice (*P*<0.01 for ileus group versus normal mice; *P*<0.05 for ileus group versus sham operated mice) (Fig. 3C and 3D). No differences were determined between *CB1–/–* and corresponding WT groups (Fig. 3A–3D).

**Figure 4:**
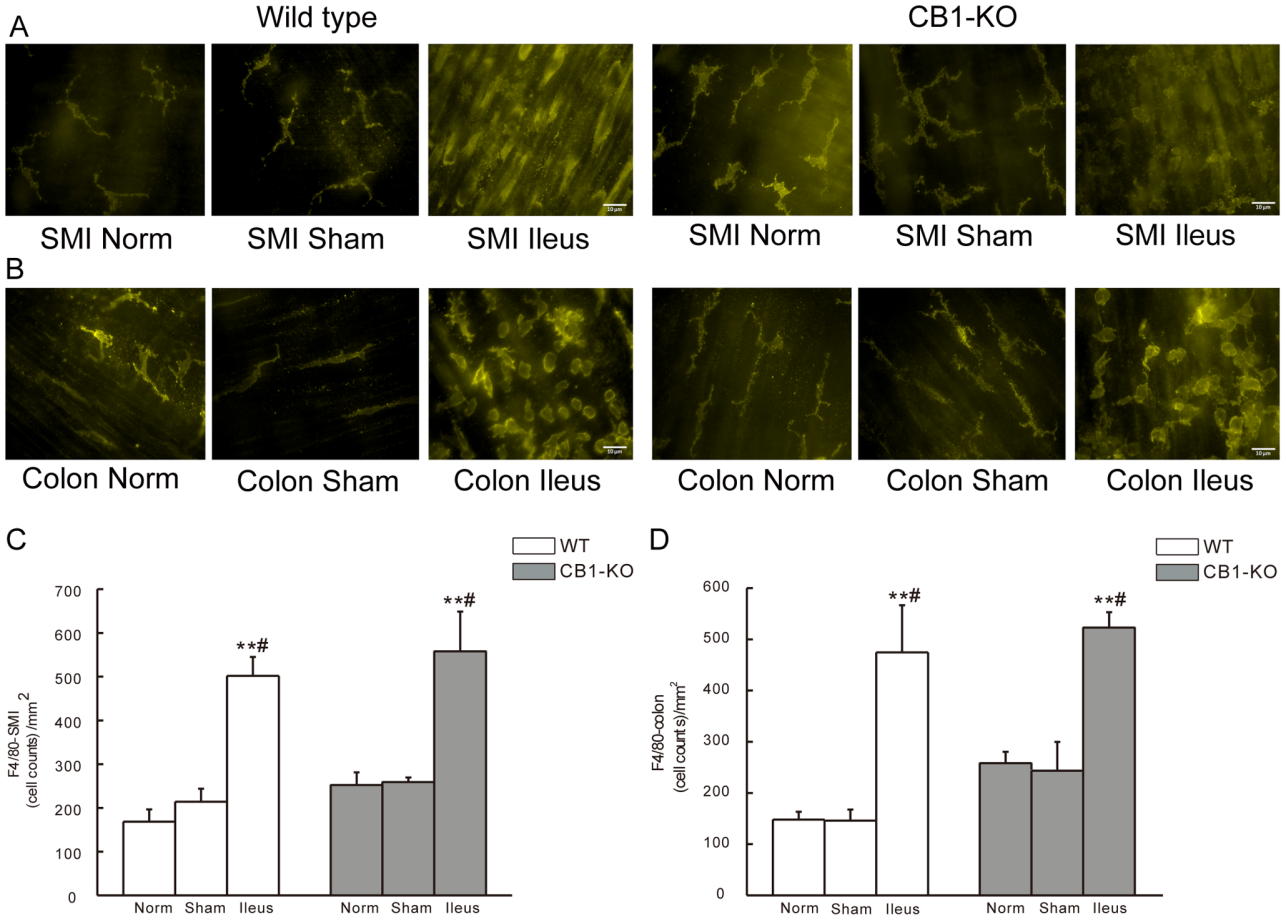
F4/80 staining for macrophages in whole mounts of intestinal muscularis of mice. A and B show representative images of F4/80 positive cells in small intestine (SMI) (A) and in colon (B) from WT or *CB1–/–* mice. C and D show statistical histograms of F4/80 positive cells in SMI (C) and in colon (D). Cell counts are given as positive cells per square millimeter (mean ± SEM, n = 6). **P<0.01 vs. normal, #*P*<0.05 vs. sham group. Scale bar  = 10 µm.

In the muscularis layer of ileum, F4/80 positive cells (i. e. macrophages) were increased per square millimeter in POI WT mice compared to normal (*P*<0.01) and sham operated controls (*P*<0.05; Fig. 4A). In *CB1–/–* mice F4/80 positive cells were similarly increased in POI animals compared to normal (p<0.01) and sham operated controls (*P*<0.05; Fig. 4C). In the muscularis layer of colon, increased numbers of macrophages were also observed during POI in both types of mice, with each group showing *P*<0.01 and *P*<0.05 vs. normal and sham controls, respectively (Fig. 4B,4D). No differences were determined between *CB1–/–* and corresponding WT groups (Fig. 4A–4D).

**Figure 5:**
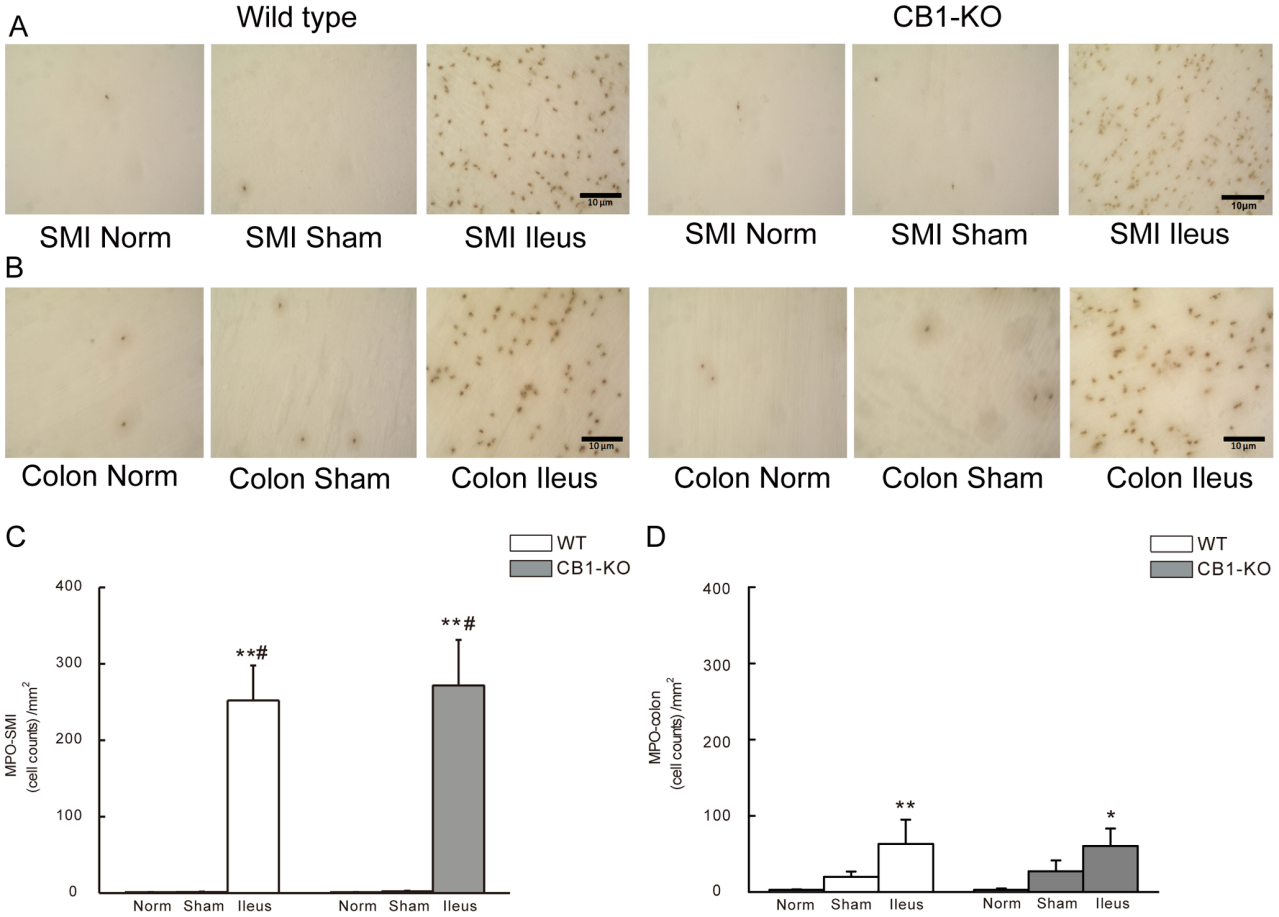
MPO-staining for neutrophils in whole mounts of intestinal muscularis of mice. A and B show representative staining figures of MPO positive cells in small intestine (SMI) (A) and in colon (B) from WT or *CB1–/–* mice. C and D show statistical histograms of MPO positive cells in SMI (C) and in colon(D). Cell counts are given as positive cells per square millimeter (mean±SEM, n = 6). *P<0.05 vs.normal, **P<0.01 vs. normal; and #*P*<0.05 vs. sham group. Scale bar  = 10 µm.

In the muscularis of ileum, MPO positive cells (i. e. monocytes and neutrophils) were increased at 24 h of POI compared to normal (*P*<0.01) and sham operated controls (*P*<0.05) in WT and *CB1–/–* mice (Fig. 5A and 5C). Cell numbers were also increased in the mucularis layer of colon in WT (*P*<0.01; Fig. 5B) and CB1–/– animals (*P*<0.05; Fig. 5D) when compared to normal controls. No significant differences of the cell counts were found between the normal and sham-operated controls in both kinds of mice (Fig. 5A–5D).

Overall, with the above-mentioned techniques, no differences of the inflammatory cell counts were found in the identically-treated groups between WT and CB1-deficient mice (Fig. 3, 4, 5).

### Plasma Levels of KC, MCP-1, IL-6 and TNF-α

**Figure 6:**
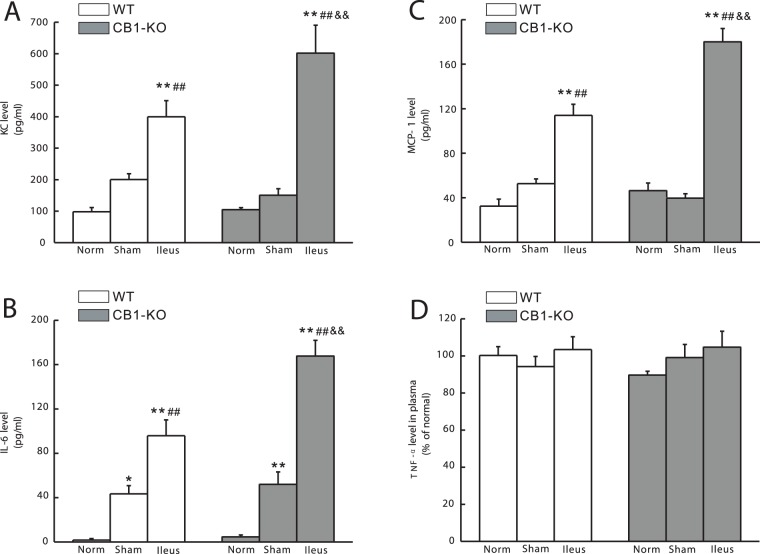
Levels of inflammatory chemokines and cytokines in mouse plasma. A–B show the statistical histograms of cytokine-induced neutrophil chemoattractant-1 (CINC-1/KC) and monocyte chemoattractant protein-1 (MCP-1), C–D show the statistical histograms of interlukin (IL)-6 and tumor necrotic factor (TNF)-α. The data are expressed as pg/ml plasma (A–C) or as percentage of the control (D) (mean±SEM, n = 6).*P<0.05 vs.normal, **P<0.01 vs.normal; ##*P*<0.01 vs.sham; and && *P*<0.01 vs. the identically-treated groups in WT mice.

To further investigate systemic inflammation, plasma levels of KC, MCP-1, IL-6, and TNF-α were evaluated. In ileus animals, levels of KC, MCP-1 and IL-6 were elevated at 24 h of POI, in both WT and *CB1–/–* mice as compared to corresponding normal or sham control groups (*P*<0.01; Fig. 6A–6C). *CB1–/–* mice showed higher plasma levels of these chemokines and cytokines when compared with WT-mice in identically treated ileus groups (*P*<0.01; Fig. 6A–6C). IL-6 levels were significantly increased even in the sham operated groups when compared with that in the normal controls in WT mice (*P*<0.05) and *CB1–/–* mice (*P*<0.01). TNF-α level remained unchanged among all experimental groups (Fig. 6D).

### p38MAPK Expression in Ileum and Colon

**Figure 7:**
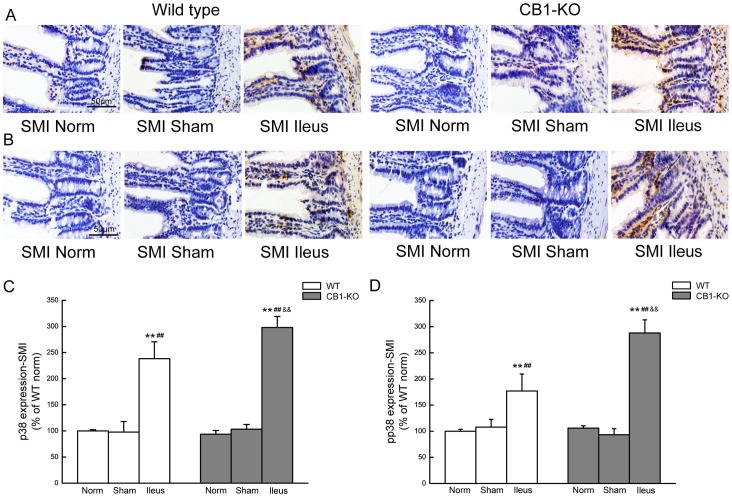
p38MAPK expression in the ileum of mice. A and C show the representative images and summarizing histograms of p38 in mouse ileum, and B and D show pp38 in mouse ileum of WT and *CB1–/–* mice. Data are given as mean ± SEM (n = 4/group). **P<0.01 vs.normal, ##*P*<0.01 vs.sham, and && *P*<0.01 vs. the identically-treated groups in WT mice. SMI means small intestine. Scale bar  = 50 µm.

Representative images and summarizing histograms for p38 and its phosphorylated form (pp38) in ileum of wild-type and *CB1–/–* mice were shown in Fig. 7. p38 expression is shown in Fig. 7A and 7C, and pp38 in Fig. 7B and 7D. It was noted that p38 and pp38 were lightly expressed in the mucosal epithelium and submucosa of ileum in normal or sham operated mice. During POI, p38 and pp38 expressions were significantly enhanced (*P*<0.01) and the expressions were particularly located in immune cells in the submucosal plexus and in smooth muscle layer (Fig. 7A and 7B). The enhancement of p38 and pp38 expression was even more obvious in *CB1–/–* mice during POI compared to the corresponding WT group (*P*<0.01; Fig. 7C and D).

**Figure 8:**
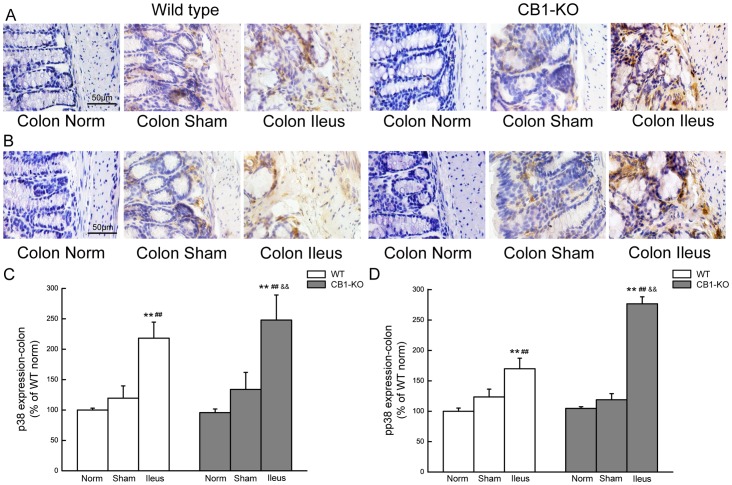
p38MAPK expression in the colon of mice. A and C display images and histograms for p38 in colon, and B and D for pp38 in colon of WT and *CB1–/–* mice. Data are given as mean±SEM (n = 4/group). *P<0.05, **P<0.01 vs.normal; ##*P*<0.01 vs.sham; and && *P*<0.01 vs. the identically-treated groups in WT mice. Scale bar  = 50 µm.

Representative expression images and summarizing histograms for p38 and pp38 in colon of both kinds of mice are given in Fig. 8, as p38 expression is shown in Fig. 8A and 8C, and pp38 in Fig. 8B and 8D. Similar change patterns as that in the ileum were found. During POI, p38 and especially pp38 expressions were enhanced in both kinds of mice (*P*<0.01), with more significant increase in *CB1–/–* mice (*P*<0.05 for p38 or *P*<0.01 for pp38 vs. the corresponding WT groups(Fig. 8C and 8D).

## Discussion

Pathogenesis of POI involves a number of factors, such as anesthesia, surgical access trauma, and intestinal manipulation, which lead to neuro-humoral dysregulation of GI motility [3]–[6]. In late 1990 s, an inflammatory response was described during POI and subsequently confirmed by numerous reports [7], [8]. At present, it is generally accepted that development of POI consists of two stages [10]. The first stage is the early postoperative period, within three hours after an abdominal operation, during which the spinal nerve pathway triggers nerve reflex inhibition of GI motility: spinal afferent nerve fibers are sensitized probably by events such as tissue damage, ischemia, or inflammation of potentially harmful mechanical/chemical stimuli resulting from surgery [27]. The second phase of POI seems to involve primarily the inflammatory response characterized by migration of leukocytes into the intestinal muscularis layer, and the release of pro-inflammatory cytokines, resulting in longer inhibition of GI motility. Specifically, POI is characterized by infiltration of leukocytes in the gut wall 24 h after surgery, and the majority of these cells are macrophages [7], [10], [28]. In this study, the method we used to induce postoperative ileus in mice is by mechanical intestinal manipulation which is an extremely well-established model world-wide [9], [29], [30]. However, it needs to be controlled by group size and standardized use under defined circumstances, to avoid the variability in experimental groups. In addition, we set up a time of 24 h after surgery as the point for investigation, considering that during this period the inflammation develops its full impact on inhibition of GI motility [28], [31].

The present study has showed that abdominal surgery is followed by inhibition of GI motility, despite the fact that organ structure of the GI tract remains intact and there is no significant damage in intestine, be it ileum or colon. At 24 h after surgery, animals still fail to recover from inhibition of GI motility. Our results keep in line as well with previous reports that a large number of immune cells, including macrophages, neutrophils, and mast cells infiltrate the muscularis layer of the small intestine and colon. In addition to the immune cell infiltration, many biologically active substances are released, including neutrophil chemotactic factors, proteases, IL-1, IL-8, adhesion molecules, collagenase, elastase, oxygen free radicals, and a variety of pro-inflammatory cytokines secreted by neutrophils. Furthermore, mast cell mediators such as histamine and 5-serotonin are released as well during the process. All these substances serve as inflammatory mediators, triggering a local and systemic inflammatory response directly and/or indirectly, and interfering with the motility of GI tract [32], [33]. In addition to previous reports on the immune response during POI, we have observed in this study the elevated levels of plasma IL-6, monocyte chemoattractant protein-1 (MCP-1), and cytokine-induced neutrophil chemoattractant-1 (CINC-1/KC), as well as increased expression of p38MAPK and its phosphorylated form pp38 which proves the activation of p38. As inflammation develops, POI deteriorates.

Cannabinoid receptor-1 (CB1) is a well-characterized cannabinoid receptor. Like its sib cannabinoid receptor-2 (CB2), it is a G-protein coupled membrane receptor and a primary target for endogenous cannabinoids [34], [35]. CB1 receptors are mainly distributed in brain tissue, thyroid, adrenal gland, liver cells, vascular endothelial cells, and GI tract [11]–[15]. The data from Casu MA, et al. demonstrated that intense positive CB1 receptor immunoreactivity exists in the all regions of the gut of mouse [13]. In the GI tract, the cannabinoid system is involved in regulation of motility, secretion, sensation and inflammation. Several studies have showed that stimulation of CB1 receptors slows motility throughout the gut, including small intestinal and colonic transit [19], [20]. Immunohistochemical studies have demonstrated that neurons which express CB receptors in the GI tract are typically cholinergic, and that activation of CB receptors inhibits the release of acetylcholine with subsequent slowing of intestinal motility and delay in gastric emptying [36]. Cannabinoids such as ANA, THC, and HU210 have the potential to activate mouse intestinal CB l receptors with subsequent inhibition of GI motility [37]; whereas CB1 receptor inhibitors promote bowel movements, increase GI transit, and counteract lipopolysaccharides (LPS)-induced GI motility disorders [23], [38], [39]. In this report, we provide further experimental evidence that the GI transit movement in the sham group or in the normal group of *CB1–/–* mice is faster than that of wild-type mice, confirming the inhibitory effect of CB1 on GI motility. It is worthy of note that inhibition of GI motility in *CB1–/–* mice is similar to that in WT animals, suggesting that the CB1 receptor and its ligands are not involved in the motility disorder during POI. However, it has been found that in either ileal or colonic smooth muscle layers of *CB1* knockout mice, there is obvious infiltration of immune cells with the detection of increased numbers of mononuclear macrophages, neutrophils, and mast cells. Although the numbers of these inflammatory cells are well-matched to those in wild–type groups, the plasma levels of IL-6, MCP-1, CINC-1/KC increase in *CB1–/–* mice, suggesting activation and increased secretion of these inflammatory mediators by immune cells in *CB1–/–* mice.

As regards the interaction between the CB system and inflammation, controversial reports exist. Most of them support the concept that et al cannabinoids have anti-inflammatory function. For example, Massa F, [24] used different chemicals to induce experimental bowel inflammation, and found that colitis had been more severe in CB1 knockout mice compared to that in wild type mice. Moreover, the pretreatment of wild type mice with CB1 receptor antagonists gave rise to an up-regulated inflammatory response, while CB1 receptor agonists subdued inflammatory response [40]. Other studies also elucidated that endogenous ligands of CB1 receptors, such as anandamide (AEA), 2-arachidonoylglycerol (2–AG), and palmitoylethanolamide (PEA) were found capable of attenuating inflammation through decreasing proinflammatory cytokine release, inhibiting mast-cell degranulation and reducing edema [22], [23], [41]. Our present results suggest that the anti-inflammatory action of CB1 in the inflammatory reaction of POI might result from the inhibition of cytokine release, but is independent of immune cell numbers.

The mechanisms of the anti-inflammatory effects of cannabinoids on gastrointestinal inflammatory diseases are far from elucidated. It is not clear whether the p38MAPK signaling pathway, the cholinergic anti-inflammatory pathway, or opioids and other systems are involved. CB1 and CB2 receptors were shown to be involved in the regulation of the mitogen-activated protein kinases (MAPKs), including the extracellular signal-regulating kinase 1 and 2 (ERK1/2), p38 MAPK and c-Jun N-terminal kinase (JNK). Activated p38MAPK increases the recruitment of immune cells, activates lymphocytes and neutrophils and delays the apoptosis of these cells [42], [43], which are the major source of perpetual production of the inflammatory mediators. The present study shows an elevated expression of p38 and its phosphorylated form (pp38) in the intestinal tissues at 24 h POI, and this phenomenon is more pronounced in CB1–/– animals. Taken together with the significantly elevated plasma levels of inflammatory mediators in CB1–/– mice, the results suggest that p38 MAPK actively participate in the local and systematic inflammatory response during POI. In addition, CB1 receptors are involved in anti-inflammatory regulation by erasing or inhibiting the p38 MAPK activation.

In summary, this study has demonstrated in a mouse POI experimental model, that GI transit is reduced in *CB1–/–* animals and their wild-type controls. In both types of mice, the inflammatory response which accompanies POI was similar when analyzing the cellular level i. e. immune cell infiltration. However, several chemokines and cytokines gauging the systemic inflammatory response were elevated in *CB1–/–* animals compared to wild-type controls. In conclusion, although absence of the CB1 receptor did not alter inhibition of GI motility during postoperative ileus, the anti-inflammatory effect was weakened by which POI may be promoted and developed. Hence, CB1 receptor activation rather than inhibition may reduce the inflammatory response in POI, which has a remote potential to relate into reduced inhibition of intestinal motility during POI.
